# Retraction Note: CO_2_ mediated optical investigation of pyridine transformation into 2,2′-bipyridine for nitrogen pollutant removal

**DOI:** 10.1038/s41598-026-59174-7

**Published:** 2026-06-22

**Authors:** Mohammed El-Amine Nouairi, Mohammed Freha

**Affiliations:** 1 Faculty of Sciences & Technology, Department of Science and Technology, University Mustapha Stambouli of Mascara, Mascara, Algeria; 2 Process Engineering and Solution Chemistry Laboratory (LGPCS), University Mustapha Stambouli of Mascara, Mascara, Algeria

Retraction of: *Scientific Reports* 10.1038/s41598-025-31831-3, published online 12 December 2025

The Editors have retracted this Article.

After publication, concerns were raised about the authorship of this paper. Despite repeated requests, the Editors were unable to verify the authorship of this Article, or obtain the raw data for the research reported from the Authors. The Editors have therefore lost confidence in the integrity of the Article.

Mohammed El-Amine Nouairi disagrees with the retraction. Mohammed Freha did not respond the correspondence from the Editors about this retraction.

